# Genetic aetiologies in relation to response to the ketogenic diet in 226 children with epilepsy

**DOI:** 10.1093/braincomms/fcaf134

**Published:** 2025-04-05

**Authors:** Maria Dahlin, Tommy Stödberg, Elin Ekman, Virpi Töhönen, Anna Wedell

**Affiliations:** Neuropaediatric Unit, Department of Women’s and Children’s Health, Karolinska Institutet, Stockholm 171 77, Sweden; Neuropediatric Department, Astrid Lindgren Children’s Hospital, Karolinska University Hospital, Stockholm 171 76, Sweden; Neuropaediatric Unit, Department of Women’s and Children’s Health, Karolinska Institutet, Stockholm 171 77, Sweden; Neuropediatric Department, Astrid Lindgren Children’s Hospital, Karolinska University Hospital, Stockholm 171 76, Sweden; Centre for Inherited Metabolic Diseases, Karolinska University Hospital, Stockholm 171 76, Sweden; Centre for Inherited Metabolic Diseases, Karolinska University Hospital, Stockholm 171 76, Sweden; Department of Molecular Medicine and Surgery, Karolinska Institutet, Stockholm 171 77, Sweden; Centre for Inherited Metabolic Diseases, Karolinska University Hospital, Stockholm 171 76, Sweden; Department of Molecular Medicine and Surgery, Karolinska Institutet, Stockholm 171 77, Sweden

**Keywords:** ketogenic diet, genetic causative variants, WGS, epilepsy, seizures

## Abstract

A ketogenic diet is used in children with drug-resistant epilepsy but predictors for efficacy are largely lacking. Our aim was to evaluate if causative genetic variants could predict seizure response to the ketogenic diet. A cohort study of 226 children with refractory epilepsy and classic ketogenic diet treatment for at least 3 months (76.9% of the 294 who started) was performed. The median age at diet start was 5.1 years (range 0.1–17.8), 118 were girls and 108 boys. They had previous trials of a median of 6.0 anti-seizure medications (range 0–12) and intellectual disability was found in 87%. Seizure response (≥50% reduction) was found in 138/226 patients (61.1%) at 3 months, 121 (53.5%) at 6 months, 107 (47.3%) at 1 year and in 80 (37.0%) at 2 years follow-up of ketogenic diet. Age of epilepsy onset was lower and combined epilepsy type less common in responders compared to non-responders but no differences were found for specific seizure types, ketogenic ratio or beta-hydroxybutyric acid blood levels. A causative pathogenic/likely pathogenic variant was detected in 107/153 = 69.9% in 48 different genes. Next generation sequencing was used in 91/226 (40%) cases with a diagnostic yield of 58.2% (53/91). In comparison with cases without a revealed genetic aetiology, patients with a causative genetic variant had less atonic seizures and epileptic spasms and a better seizure response with 17.3% seizure free and 25% with >90% seizure reduction at 2-year follow-up. Causative variants in *SLC2A1*, *SCN1A*, *STXBP1* and *PAFAH1B1* showed significant diet response (*P* < 0.05) and good efficacy was also associated with *DEPDC5*, *GLDC*, *KCNT1*, *PDHA1*, *SLC25A12* and *TSC1*. Causative variants in *COL4A1* and *DYNC1H1* were among genes linked to a lack of response. To our knowledge not described previously, we report a good ketogenic diet response related to causative variants in *CSNK2A1*, *FARS2*, *GABRB3*, *GRIN1*, *KCNA2*, *KCTD3*, *STX1B* and *SLC16A2* but a lack of response for causative variants in *CLN5*, *GLI3*, *MACF1*, *MAGEL2*, *NANS*, *NEMO/IKBKG*, *RORB*, *SLC17A5* and *UFSP2.* After grouping of genes into functional groups, causative variants in transporter genes had the best response (*P* = 0.009) and variants in other membrane-related proteins (ion channels and neurotransmitter receptors) also showed good efficacy. However, the gene group related to cell structural integrity and/or homeostasis had the worst diet response (*P* = 0.00006). In conclusion, our results support that causative genetic variants may be used as prognostic markers of ketogenic diet response, constituting an example in the expanding area of precision medicine.

## Teaser

Dahlin *et al*. studied 226 children with refractory epilepsy treated with ketogenic diet, aiming to identify genetic predictors of seizure response. Causative variants were found in 48 genes and those related to transporters, ion channels and neurotransmitters were associated with efficacy, while genes linked to cell structural integrity were not.

## Graphical Abstract

**Graphical Abstract:**
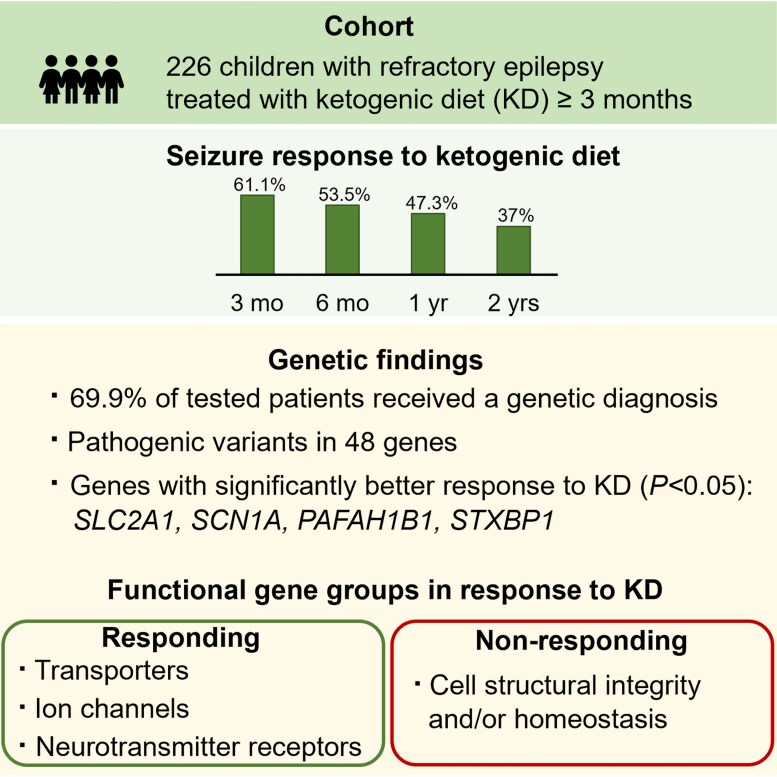

## Introduction

The majority of children with epilepsy obtain seizure freedom by using anti-seizure medications (ASMs). However, one third have a drug-resistant epilepsy which is defined as failure to achieve seizure freedom following adequate trials of two tolerated and appropriately chosen ASMs.^1^ In this group, the ketogenic diet (KD) is a well-established treatment. Studies show that about half of the children on the diet get a seizure reduction of ≥50% and up to 10% achieve seizure freedom.^2-4^

Data are emerging on beneficial effects of KD in some neurometabolic disorders in which epilepsy is common.^5^ These include glucose transporter protein 1 deficiency syndrome (Glut1 DS), pyruvate dehydrogenase complex (PDHC) deficiency and recently in aspartate-glutamate carrier isoform 1 (AGC1) deficiency^6,7^ and non-ketotic hyperglycinemia.^8^ In these conditions, improvements may be seen not only concerning seizures but also in cognitive and motor functions.^9,10^ Epilepsy due to pathogenic variants in other, not yet identified genes involved in metabolism may have a good treatment response to KD.^5^

A retrospective study from South Korea of early onset epileptic encephalopathy showed that treatment response to drugs as well as KD varied according to genetic aetiology.^11^ In another study, 155 children treated with KD were examined with a gene panel of 72 genes for epileptic encephalopathy and a pathogenic variant was verified in 73 children (47.1%).^12^ Causative variants in *STXBP1* were associated with a high responder rate while variants in other genes had a lower KD response. The Mayo Clinic reported 56 children with genetic epilepsy and KD.^13^ Conclusions were that some variants gave a better KD response and that KD was effective in refractory epilepsy of genetic origin.

In order to investigate whether pathogenic gene variants may be prognostic markers of treatment response, we examined the response to KD in relation to genetic aetiology in a large cohort of children with drug-resistant epilepsy treated at a single centre during a 25-year period.

## Materials and methods

### Patients

The study was performed at the Karolinska University Hospital, Stockholm, as part of an investigation on the efficacy and safety of KD in children. It includes children (0–17.9 years) who started the classical KD between 1995 and February 2023 due to epilepsy. The patients were enrolled as they attended the Epilepsy Outpatient Clinic and included regional as well as referral patients although regional patients followed at our clinic until 18 years comprised the majority. The decision to start the KD was based on clinical indications. KD was offered if epilepsy was drug-resistant and epilepsy surgery had been considered and in epilepsy in children with a neurometabolic disorder in which KD is recommended including Glut1 DS and PDHC deficiency. Children, who stopped KD before follow-up visit at 3 months on diet, when efficacy was evaluated, were excluded from the study.

### Study design

Clinical data retrieved from medical records included gender, age at KD initiation, age at epilepsy onset, epilepsy type and seizure type(s), use of ASMs and presence of a gastrostomy. The type of epilepsy as well as seizure(s) were defined in accordance with the International League Against Epilepsy new classification systems.^14,15^ The epilepsy types were classified into focal, generalized or combined. Non-motor seizures with generalized onset, including typical and atypical absences, were not included as they are difficult to count. Spasms could not always be classified into focal or generalized and are therefore classified only as epileptic spasms. This also applies to tonic, atonic and myoclonic seizures. Detailed EEG data and epilepsy syndrome features were not always available and therefore epilepsy syndromes were not classified. The number of previously used ASMs as well as ASMs at diet start were compiled. Clinical data concerning intellectual disability (ID) and motor dysfunction at diet start were collected. In young children, not yet tested at diet start, demographic data on intellectual function was retrieved at a later time point when neuropsychological testing had been performed. Evaluation of cognitive level and motor performance followed clinical routines; see details in Supplementary Materials.

The aetiology of the epilepsy was assessed by review of the medical history including clinical examinations and results from neurophysiological, neuroradiological, metabolic and genetic investigations.

Seizure response was calculated from seizure calendars kept by parents and the mean seizure frequencies the month before KD start and the month before each regular follow-up visit were compared. Children with a seizure reduction of ≥50% were considered responders and those with less non-responders. Data on patients with a seizure reduction of >90% and seizure freedom were also collected.

Ethical approval was obtained from the Swedish ethical review board, DNR 2008/351-31, 00–187 and 2014/1177-32 and informed consent was obtained from parents and, when possible, from patients.

### Genetic investigations

Children without a probable aetiology of their epilepsy were offered whole exome (WES) and/or whole genome sequencing (WGS) once these methodologies became available. Children who started KD before this era were offered the genetic testing methods that were available at the time and some were later offered WES/WGS as part of this study.

WGS for diagnosis of rare diseases, including epilepsy, was implemented at our centre in 2015 as part of Genomic Medicine Center Karolinska-Rare Diseases, an academic-clinical partnership between the Clinical Genomics facility at the Science for Life Laboratory and three specialized clinics, including the Centre for Inherited Metabolic diseases, Clinical Genetics and Clinical Immunology (Supplementary Materials). Preceding the implementation of WGS, genetic investigations were conducted with WES from 2014. The WES/WGS data was analysed using precompiled, regularly updated gene lists, with additional HPO-generated gene lists in certain cases, as previously described.^16^ All results were reviewed by a multi-disciplinary team. Comparative genomic hybridization (array CGH) has been employed since 2002 and Gene-specific PCR and Sanger sequencing since 1998 when clinical symptoms indicate a mutation in a specific gene. Other methods used in some older cases included QF-PCR, FISH, karyotyping and MLPA. If a pathogenic cause was identified using these methods WES/WGS was not run. Genomic DNA was extracted from blood samples and for most cases where parental samples were available a trio analysis was performed.

### Initiation and maintenance of the ketogenic diet

The procedures on how to initiate and maintain the KD at our centre is described in detail in a previous paper^17^ as well as in Supplementary Materials. To initiate and maintain the KD, we followed a standardized protocol for the classic KD a slightly modified version of the protocol from the Johns Hopkins Hospital.^18^ A dietitian calculated the daily calorie level, the KD ratio and the composition of all meals and supplements for each child. The KD was started during an inpatient stay to introduce meals, monitor side effects and carry out an educational programme. Follow-up outpatient visits were held at 3 months for evaluation of seizure frequency and behaviour and after 6 months and 1 and 2 years for evaluation of seizure frequencies. At all visits, blood ketone beta-hydroxybutyrate (β-OHB) was checked to evaluate treatment.

### Statistical analysis

Descriptive statistics were presented as median and range and often also mean and standard deviation (SD) for continuous variables, and as counts and proportions (%) for ordinal and nominal variables. The independence of descriptive statistics across outcome groups (seizure response and genetic aetiology) was tested using the χ^2^ test or the Mann–Whitney U-test, as appropriate.

Outcomes were assessed using univariate analysis, with proportions calculated for non-continuous variables. Relative risk (RR) estimates were calculated for all variables. For both outcome proportions and RR, 95% confidence intervals were constructed. In the univariate analysis of RR for seizure type, all seizure types were included in the same model. This approach was taken because there was no unexposed group and multiple seizure types could occur in the same patient. Variables with a univariate *P*-value of <0.05 for RR were included in a final multi-variable model. All RR’s were estimated using log-binomial regression.^19,20^ Likelihood-ratio tests were used to calculate *P*-values and to construct 95% confidence intervals for both outcome proportions and RR. *P*-value from odds ratios (OR) was used to assess independence of the frequency distribution of disease-causing variants among responders and non-responders. All statistical analyses were performed using R software (version 4.4.1, R Foundation for Statistical Computing, Vienna, Austria).

## Results

### Patients

**Figure 1:**
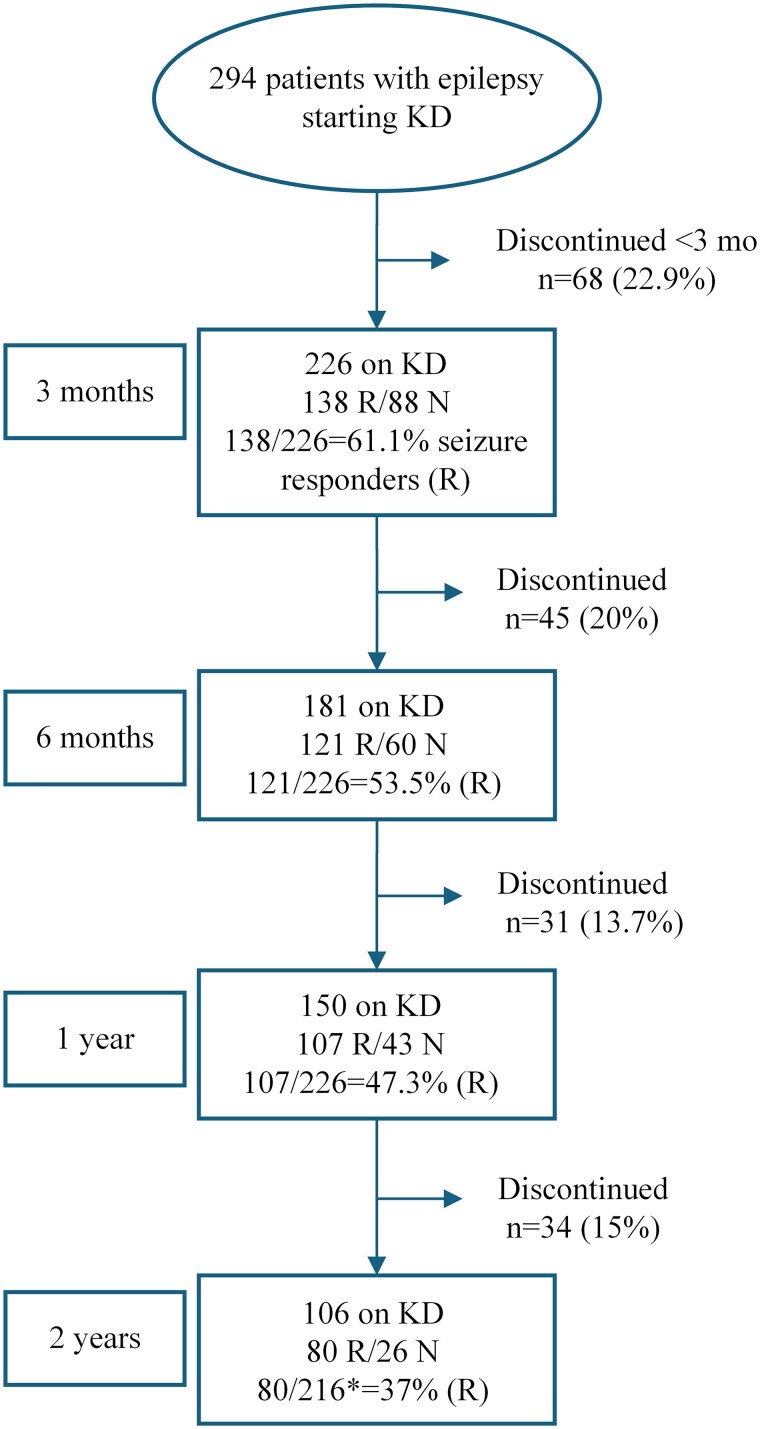
**Flowchart showing seizure response for the total cohort of children with epilepsy (*n* = 294) starting on the classic KD at our centre.** 226 children completed 3 months on diet and constitute the study cohort. Data on seizure response at follow-up after 3 and 6 months and 1 and 2 years on diet are shown. Those with seizure reduction ≥50% are considered responders (R) and <50% reduction as non-responders (N). Discontinuation of KD was due to inefficacy, side effects and/or noncompliance. *10 patients shorter follow-up than 2 years since the start of KD.

A total of 294 patients initiated the classic KD due to epilepsy at our centre during 1995–2023 (Fig. 1). However, 68 (23.1%) patients discontinued the diet before the 3-month follow-up, due to lack of compliance in 56 cases and side effects in 12 (three vomiting, three increased number of seizures, two hypoglycaemia, one each of somnolence, obstipation, hyponatremia, increased cholesterol). None of the side effects were serious, and all vanished after tapering the diet.

Consequently, 226 children with a 3-month follow-up were included in this study (Table 1, first section). The median age at KD start was 5.1 years and range 1 month-17.8 years (mean 6.4 ± 4.3 SD). The gender distribution comprised 118 girls and 108 boys. No difference was observed when comparing age at KD start based on gender. The age of epilepsy onset was median 0.6 years; range 0–12.7 (mean 1.5 ± 2.3 SD). The epilepsy type was generalized in 146 children, focal in 50, and combined generalized and focal in 30. Most of the children experienced multiple seizure types, with a median of 2.0; range 1–4 (mean 1.9 ± 0.8 SD) seizure types per patient. The most common seizure types were tonic seizures, found in 102 patients, followed by focal seizures with/without impaired awareness in 80.

**Table 1:** Demographic and clinical data

		Data in response to KD				Data in relation to genetic findings		
	Total cohort	Non-responders	Responders			Group 1	Group 2	Group 3
		<50%	≥50%	>90%	100%	Causative variant	No causative variant	No genetic testing
	*n =* 226	*n =* 88 (38.9%)	*n =* 138 (61.1%)	*n =* 53 (23.5%)	*n =* 28 (12.4%)	*n =* 107	*n =* 46	*n =* 73
Age at KD start y; median (range)	5.1 (0.1–17.8)	6.5 (0.1–17.8)	4.8 (0.1–17.8)	4.7 (0.2–15.3)	4.9 (0.4–15.3)	4.6 (0.1–17.8)	5.0 (0.4–17.8)	6.7 (0.7–17.8)
Sex (m/f)	108/118	42/46	66/72	24/29	11/17	49/58	22/24	37/36
Age at epilepsy onset, median	0.6 (0–12.7)	0.6 (0–12.5)	0.6 (0–12.7)	0.7 (0–7.5)	0.8 (0.1–7.5)	0.5 (0–12.0)	1.1 (0.1–12.7)	0.8 (0–12.5)
Epilepsy type no (%) Gen	146 (64.6%)	53 (36.3%)	93 (63.7%)	35 (24.0%)	17 (11.6%)	69 (47.3%)	35 (24.0%)	42 (28.8%)
Focal	50 (22.1%)	13 (26.0%)	37 (74.0%)	16 (32.0%)	9 (18.0%)	23 (46.0%)	5 (10.0%)	22 (44.0%)
Combined	30 (13.3%)	22 (73.3%)	8 (26.7%)	2 (6.7%)	2 (6.7%)	15 (50.0%)	6 (20.0%)	9 (30.0%)
No. seizure types, median	2 (1–4)	2 (1–4)	2 (1–4)	2 (1–4)	2 (1–4)	2 (1–4)	2 (1–4)	2 (1–4)
Seizure types, no (%) FAS/FIAS	79 (34.9%)	33 (41.8%)	46 (58.2%)	21 (26.6%)	14 (17.7%)	40 (50.6%)	10 (12.7%)	29 (36.7%)
FBTCS	29 (12.8%))	13 (44.8%)	16 (55.2%)	8 (27.6%)	2 (6.9%)	8 (27.6%)	3 (10.3%)	18 (62.1%)
GTC	61 (27.0%)	28 (45.9%)	33 (54.1%)	13 (21.3%)	7 (11.5%)	33 (54.1%)	10 (16.4%)	18 (29.5%)
Spasms	24 (10.6%)	11 (45.8%)	13 (54.2%)	4 (16.7%)	0	8 (33.3%)	10 (41.7%)	6 (25.0%)
Tonic	102 (45.1%)	42 (41.2%)	60 (58.8%)	17 (16.7%)	9 (8.8%)	58 (56.9%)	14 (13.7%)	30 (29.4%)
Atonic	39 (17.3%)	15 (38.5%)	24 (61.5%)	10 (25.6%)	6 (15.4%)	11 (28.2%)	20 (51.3%)	8 (20.5%)
Myoclonic	48 (21.2%)	19 (39.6%)	29 (60.4%)	9 (18.8%)	5 (10.4%)	26 (54.2%)	6 (12.5%)	16 (33.3%)
No. previous ASMs, median	6.0 (0–12)	6 (0–12)	6 (0–12)	6 (0–9)	6 (0–9)	6 (0–12)	6 (3–11)	6 (3–12)
No. concomitant ASMs, median	2.0 (0–4)	2 (0–4)	2 (0–4)	2 (0–4)	2 (0–4)	2 (0–4)	3 (1–4)	2 (0–4)
ID no (%)	198 (87.6%)	80 (40.4%)	118 (59.6%)	42 (21.2%)	20 (10.1%)	98 (49.5%)	37 (18.7%)	63 (31.8%)
Motor dysfunction, no (%)	111 (49.1%)	41 (36.9%)	70 (63.1%)	21 (18.9%)	7 (6.3%)	59 (53.2%)	19 (17.1%)	33 (29.7%)
Gastrostomy, no (%)	69 (30.5%)	25 (36.2%)	44 (63.7%)	15 (21.7%)	8 (11.6%)	46 (66.7%)	10 (14.5%)	13 (18.8%)
KD ratio 3 mo, median	3.5 (1.5–4)	3.5 (2–4)	3.5 (1.5–4)	3.5 (1.5–4)	3.3 (1.5–4)	3.5 (1.5–4)	3.5 (2–4)	4 (2–4)
4:1	103	42	59	20	11	43	15	45
3.5:1/3:1	42/43	18/17	25/27	8/10	3/3	21/19	11/11	10/13
2.5:1/2:1	21/15	7/4	14/11	7/6	4/5	13/9	5/4	3/2
** **1.5:1	2	0	2	2	2	2	0	0
β-OHB 3 mo (*n* = 217), median	4.1 (0.4–7.7)	4.1 (0.4–7.7)	4.2 (0.4–6.9)	4.8 (0.4–6.9)	4.3 (0.4–6.6)	4.3 (0.4–7.1)	3.8 (0.4–7.7)	4.1 (0.9–6.9)

Left section shows data in the total cohort (*n* = 226). In the middle section, non-responders were defined as seizure reduction <50% and Responders ≥50%. The 138 responders were thereafter sub-grouped into those with >90% and 100% response. The last section shows the total cohort classified in three groups in relation to genetic findings. Group 1: causative variant found after genetic testing with WES/WGS and/or other genetic analysis, Group 2: no causative variant found after WES/WGS, and Group 3: WES/WGS not performed.

Gen, generalized; FAS, focal aware seizures; FIAS, focal impaired awareness seizures; FBTCS, focal to bilateral tonic-clonic seizures; GTC, generalized tonic-clonic seizures; Spasms, epileptic spasms; ASMs, anti-seizure medications; β-OHB, beta-hydroxybutyric acid.

Five patients with epilepsy had not been treated with ASMs before KD start as seizure onset appeared simultaneously with investigations that revealed a neurometabolic disorder in which KD is recommended (four patients with PDHC deficiency and one patient with Glut1 DS). In these patients KD was initiated rapidly and before starting ASMs. Additionally, four patients with epilepsy and Glut1 DS had taken only one ASM before diet start. Thus, 217 out of 226 (96%) patients had a drug-resistant epilepsy.^1^ Before diet initiation, they had tried a median number of 6.0 ASMs; range 0–12 (mean 6.2 ± 2.4 SD). Two patients tapered the ASMs before diet start due to lack of efficacy (previous trials of nine and five ASMs, respectively). Thus, at diet start, 219 children were concurrently taking ASMs with a median number of 2.0 ASMs per child.

The most commonly used ASMs were valproate and clobazam (*n* = 93 patients each), topiramate (*n* = 63), lamotrigine (*n* = 51), levetiracetam (*n* = 46), clonazepam (*n* = 32) and zonisamide (*n* = 20).

ID was noted in a large majority, 198 (87.6%) children, and motor disability in about half, 111 (49.1%). Many children were hypotonic and had swallowing difficulties and during recent years gastrostomy has become more frequently used. In this cohort, 69 (30.5%) children had a permanent gastrostomy.

The ratio at 3 months on KD for the 226 patients varied between 4:1 and 1.5:1 with a median ratio of 3.5 (mean 3.4:1 ± 0.7 SD). The blood β-OHB level at 3 months on diet was available in 217 (96%) patients and the median level was 4.1 and the range 0.4–7.7 (mean 4.1 ± 1.5 SD) mmol/L.

### Demographic and clinical data in relation to seizure response

Demographic data in relation to KD response is presented in Table 1. Comparing responders (≥50% seizure reduction) to non-responders after three months of KD, no significant difference was found concerning age at KD start (*P* = 0.055) but a trend towards younger age was observed in responders (Table 2). No differences in response in relation to sex were found. However, the age of epilepsy onset was related to seizure efficacy with responders having a younger age at onset (*P* = 0.034; RR = 0.94, 95% CI 0.87–1.00).

**Table 2:** Possible predictors of efficacy of the KD at 3 months for the whole cohort (*n* = 226)

Predictors	Responders		Univariate binary regression		Multi-variate binary regression	
	*n*	% (95% CI)	RR (95% CI)	*P*-value	RR (95% CI)	*P*-value
Age at KD			0.98 (0.95–1.00)	0.055		
Sex						
Female	72/118	61.0 (52.0–69.5)	1.00 (0.81–1.23)	0.99		
Male	66/108	61.1 (51.7–70.0)	1			
Age at epilepsy onset			0.94 (0.88–0.99)	**0**.027*	0.94 (0.87–1.00)	**0**.034*
Epilepsy type						
Gen	93/146	63.7 (55.7–71.2)	1		1	
Focal	37/50	74.0 (60.8–84.8)	1.16 (9.93–1.41)	0.18	0.90 (0.71–1.47)	0.46
Combined	8/30	26.7 (13.2–44.0)	0.42 (0.21–0.70)	**0**.0002*	0.36 (0.17–0.74)	**0**.007*
No. of seizure types			0.95 (0.83–1.08)	0.46		
Seizure type^a^						
FAS/FIAS	46/79	58.2 (47.2–68.7)	0.84 (0.65–1.07)	0.17	1.18 (0.69–1.59)	0.38
FBTCS	16/29	55.2 (37.2–72.3)	0.80 (0.52–1.12)	0.20	0.97 (0.66–1.29)	0.86
Spasms	13/24	54.2 (34.6–72.9)	0.72 (0.45–1.03)	0.073	0.81 (0.51–1.15)	0.28
GTC	33/61	54.1 (41.6–66.2)	0.71 (0.52–0.94)	**0**.015*	0.82 (0.60–1.09)	0.17
Tonic	60/102	58.8 (49.1–68.1)	0.78 (0.61–1.00)	**0**.049*	0.90 (0.69–1.13)	0.39
Atonic	24/39	61.5 (45.9–75.7)	0.85 (0.62–1.13)	0.27	1.02 (0.73–1.35)	0.88
Myoclonic	29/48	60.4 (46.3–73.4)	0.92 (0.69–1.17)	0.53	1.08 (0.81–1.34)	0.59
No. of previous ASMs			0.96 (0.92–0.99)	**0**.027*	0.98 (0.95–1.02)	0.29
No. of concomitant ASMs			0.93 (0.84–1.04)	0.19		
Gastrostomy						
Yes	44/69	63.8 (52.1–74.5)	1.07 (0.85–1.33)	0.53		
No	89/150	59.3 (51.4–67.0)	1			
ID	118/198	59.6 (52.7–66.3)	0.89 (0.68–1.38)	0.55		
Motor dysfunction	70/111	63.1 (53.9–71.7)	1.07 (0.87–1.33)	0.51		
KD ratio at 3 mo			0.89 (0.77–1.04)	0.14		
β-OHB at 3 mo			0.99 (0.92–1.06)	0.78		

Univariate and multi-variate binary regression analyses. Multi-variate analysis included factors that showed statistical significance in the univariate analysis.

^a^All seizure types were modelled together also in the univariate analysis since all patients were exposed to at least one seizure type and no clear reference level exists.

^*^Significant differences (*P* < 0.05) given in bold.

Gen, generalized; FAS, Focal aware seizures; FIAS, Focal impaired awareness seizures; FBTCS, focal to bilateral tonic-clonic seizures; GTC, generalized tonic-clonic seizures; Spasms, Epileptic spasms; ASMs, anti-seizure medications; β-OHB, beta-hydroxybutyric acid; mo, months.

Focal epilepsy demonstrated the highest response rate among the epilepsy types and was proportionally more prevalent in responders, with a response rate of 37/50 (74%) although this was not significant. Combined epilepsy was significantly less common in responders with a response rate of 8/30 (26.6%) (*P* = 0.007, RR = 0.36, 95% CI 0.17–0.74). Regarding specific seizure types, all types responded to KD with atonic and myoclonic seizures having proportionally the highest response rates, while GTCS and tonic seizures showed the lowest response. Nonetheless, after adjusting for confounding factors in the multi-variate analyses, these differences did not reach statistical significance (Table 2).

The number of previous ASMs was significantly greater in non-responders compared to responders in the univariate analyses; however, the correlation was not seen in the multi-variate regression analysis. No significant differences in relation to response were found concerning the number of seizure types or number of concomitant ASMs, intellectual or motor development, KD ratio, level of β-OHB or presence of a gastrostomy (Table 2).

### Demographic and clinical data in relation to genetic testing

Patients who had a causative variant confirmed through genetic testing via WES/WGS and/or other genetic methods were classified as Group 1 (*n* = 107). Those who did not have a causative variant confirmed despite genetic work-up with WES/WGS were categorized as Group 2 (*n =* 46). Patients who did not have a genetic aetiology revealed and did not undergo WES/WGS were classified as Group 3 (*n* = 73). The reasons for no WES/WGS in Group 3 was that KD treatment was given before testing was available, the patients did not remain under the care of our clinic or that a non-genetic aetiology to the epilepsy had been found (Supplementary Table S1). Due to the heterogeneity of Group 3 and the absence of updated genetic testing in this cohort, we did not include this group in the statistical comparisons with Groups 1 and 2 concerning seizure response in relation to genetic aetiology.

Groups 1 and 2 had similar age at KD start (Table 1, last section). Group 1 had an earlier age of epilepsy onset, median 0.5 (0.01–12.0) years, compared to Group 2, median 1.13 (0.08–12.7) years), however this did not reach statistical significance (*P* = 0.094, RR = 0.94, 95% CI 0.86–1.01)

There was no difference in epilepsy type between Groups 1 and 2. Concerning seizure types, patients with spasms and atonic seizures were more frequently observed in Group 2 with significantly less chance of receiving a genetic diagnosis (*P* = 0.025 and *P* = 0.000017, respectively) (Table 3). Comparison of the number of previous and concomitant ASMs, presence of a gastrostomy, ID and motor dysfunction, mean KD ratio or the level of β-OHB revealed no difference between Groups 1 and 2 (Table 1).

**Table 3:** Genetic findings in patients with different seizure types

Seizure type	*n =* Genetic findings	%	Univariate binary regression		Multi-variate binary regression	
			RR (95% CI)	*P*-value	RR (95% CI)	*P*-value
Myoclonic	26/32	81	1.21 (0.95–1.47)	0.10	1.20 (0.99–1.36)	0.062
Tonic	58/72	81	1.33 (1.09–1.66)	**0**.0063*	1.08 (0.88–1.31)	0.42
FAS/FIAS	40/50	80	1.23 (1.00–1.50)	0.053	1.00 (0.83–1.19)	1.00
GTC	33/43	77	1.14 (0.91–1.39)	0.24	1.00 (0.82–1.21)	1.00
FBTCS	8/11	73	1.04 (0.62–1.38)	0.83	0.91 (0.55–1.31)	0.55
Spasms	8/18	44	0.61 (0.32–0.93)	**0**.016*	0.60 (0.31–0.95)	**0**.025*
Atonic	11/31	36	0.45 (0.26–0.68)	**0**.000006*	0.44 (0.25–0.68)	**0**.00002*

FAS, focal aware seizures; FIAS, focal impaired awareness seizures; GTC, generalized tonic-clonic seizures; FBTCS, focal to bilateral tonic clonic seizures; Spasms, epileptic spasms.

^*^Significant differences (*P* < 0.05) given in bold.

### Seizure response with ketogenic diet at different time points

Seizure response data were available in all 226 children at 3- and 6-months and 1-year follow-up time and for 216 children at 2-year follow-up. The missing data at 2 years consists of 10 children who had not yet reached 2-year follow-up since diet start (Table 4; Supplementary Tables S2 and S3). Response data are calculated for patients who had reached each time point, being either on diet or having discontinued KD. Seizure reduction of ≥50% (responders) was found in 138 patients (61.1%) at 3 months, 121 (53.5%) at 6 months, 107 (47.3%) at 1 year and in 80 (37.0%) at 2-year follow-up (Table 4). Seizure reduction >90% (including seizure free) was encountered in 53 patients (23.5%) at 3 months, 54 (23.9%) at 6 months, 46 (20.4%) at 1 year and in 34 (15.7%) at 2 years follow-up (Table 4). Seizure freedom was found in 28 children (12.4%) at 3 months, 31 (13.7%) at 6 months, 28 (12.4%) at 1 year and in 24 (11.1%) children at 2 years follow-up of KD (Table 4). In two patients an increased seizure frequency was noted, one patient with a variant in the *UFSP2* gene and one with unknown aetiology.

**Table 4:** Seizure response to KD at 3 and 6 months and at 1 and 2 years for the total cohort and Groups 1–3 in relation to genetic findings

Seizure reduction	3 months (*n*)	%	6 months (*n*)	%	1 year (*n*)	%	2 years (*n*)	%
**≥50%**								
Total cohort (*n* = 226)	138/226	61.1	121/226	53.5	107/226	47.3	80/216	37.0
Group 1—causative variant	68/107	63.6	62/107	57.9	57/107	53.5	46/104	44.2
Group 2—no causative variant	28/46	60.9	24/46	52.2	20/46	43.5	15/41	36.6
Group 3—no genetic testing	42/73	57.5	35/73	47.9	30/73	41.1	19/71	26.8
**>90%**								
Total cohort (*n* = 226)	53/226	23.5	54/226	23.9	46/226	29.4	34/216	15.7
Group 1—causative variant	31/107	29.0	31/107	29.0	29/107	**27**.1*	26/104	**25**.0*
Group 2—no causative variant	8/46	17.4	9/46	19.6	5/46	13.0	3/41	7.3
Group 3—no genetic testing	14/73	19.2	14/73	19.2	11/73	15.1	5/71	7.0
**Seizure free**								
Total cohort (*n* = 226)	28/226	12.4	31/226	13.7	28/226	12.4	24/216	11.1
Group 1—causative variant	19/107	17.8	23/107	**21**.5*	21/107	**19**.6*	18/104	17.3
Group 2—no causative variant	4/46	8.7	3/46	6.5	3/46	6.5	3/41	7.3
Group 3—no genetic testing	5/73	6.8	5/73	6.8	4/73	5.5	3/71	4.2

Statistical comparison of seizure reduction was done between Groups 1 and 2.

^*^Significant differences (*P* < 0.05) given in bold.

Table 4 also presents seizure response related to genetic findings in Groups 1, 2 and 3. Statistical analyses comparing seizure response between Groups 1 and 2 was performed. The group with a confirmed pathogenic variant had a slightly higher proportion of children with ≥50% seizure reduction at all time points but the differences were non-significant. A notably higher proportion of individuals with a pathogenic genetic variant achieved >90% seizure reduction and seizure freedom at all time points compared to those without a causative variant. The differences reached significance for >90% seizure reduction at 1 and 2 years (*P* = 0.020, RR = 2.49, 95% CI 1.14–6.97 and *P* = 0.010, RR = 3.42, 95% CI 1.29–13.79) as well as for seizure freedom at 6 months and 1 year (*P* = 0.015, RR = 3.30, 95% CI 1.22–13.41 and *P* = 0.029, RR = 3.01, 95% CI 1.11–12.30). Notably, the efficacy seemed to be more sustained over time in the group with causative variants compared to the other groups.

In the group without WES/WGS genetic testing (Group 3) a probable aetiology to the epilepsy was found in 45.2% of the patients, hypoxic-ischaemic encephalopathy (HIE) being the most common (Supplementary Table S1). Those with prematurity, HIE and Aicardi syndrome showed proportionally the best seizure responses at 2-year follow-up.

### Response in behaviour

At 3 months on KD behaviour showed an improvement in 132 (58.4%) out of the 226 patients. The most common changes were a better social communication, more eye contact, interest in the surroundings and relations, sounds/speech and more voluntary motor activity.

### Reasons to discontinue ketogenic diet

In 69 children there was a planned tapering of KD after 2 years of effective treatment. The main reason to discontinue the diet earlier was lack of efficacy in 90 patients, compliance problems in 10, combination of lack of efficacy and compliance in 7 and complications in 4 including kidney stone in 1, infections in 2 and anxiety in 1 (Fig. 1 and Supplementary Table S2). Four children died during KD but none was assessed to be associated with the treatment (two in infections/pneumonia/sepsis, one in a postoperative complication not connected to KD and one in sudden unexpected death in epilepsy associated with *SCN1A* mutation). One patient was lost to follow-up after 6 months on diet due to change of place of residence.

### Overall genetic findings

Genetic analysis was performed in 153 out of the total cohort of 226 children. Causative, pathogenic variants were identified in 107 patients (69.9% of tested patients, 47.3% of the whole cohort) involving 48 genes. Many patients underwent several analyses. Genetic testing with next generation sequencing was done in 91 patients (40% of the whole cohort) (WGS: *n = 70*, WES: *n* = 21) and gave a diagnostic yield of 58.2% (53/91). In 54 patients, causative findings were found using other methods (gene-specific Sanger sequencing, FISH, Karyotyping, MLPA, QF-PCR and array CGH). A summary of all genes with causative mutations and number of patients and their response regarding seizure frequency and behaviour, is presented in Tables 5 and 6, and details in individual patients in Supplementary Table S3.

**Table 5:** Disease-causing variants in individual genes in responding and non-responding patients

Gene	Patients *n*	Behaviour response 3 months; *n* (%)	Seizure response 3 months; *n* (%)	Seizure response 6 months; *n* (%)	Seizure response 1 year; *n* (%)	Seizure response 2 years; *n* (%)
**Responders (*n* = 31 genes)**						
*ADSL*	1	1/1 (100)	1/1 (100)	1/1 (100)	1/1 (100)	0/1 (0)
*BRAF*	1	0/1 (0)	1/1 (100)	1/1 (100)	1/1 (100)	0/1 (0)
*CDKL5*	3	1/3 (33)	1/3 (33)	1/3 (33)	1/3 (33)	1/3 (33)
*CSNK2A1*	1	1/1 (100)	1/1 (100)	1/1 (100)	1/1 (100)	1/1 (100)
*DEPDC5*	2	2/2 (100)	2/2 (100)	2/2 (100)	2/2 (100)	2/2 (100)
*FARS2*	1	1/1 (100)	1/1 (100)	1/1 (100)	1/1 (100)	1/1 (100)
*GABRB3*	1	0/1 (0)	1/1 (100)	0/1 (0)	0/1 (0)	0/1 (0)
*GLDC*	2	1/2 (50)	1/2 (50)	1/2 (50)	2/2 (100)	2/2 (100)
*GRIN1*	1	1/1 (100)	1/1 (100)	1/1 (100)	1/1 (100)	1/1 (100)
*GRIN2D*	1	1/1 (100)	1/1 (100)	1/1 (100)	1/1 (100)	1/1 (100)
*KCNA2*	1	1/1 (100)	1/1 (100)	0/1 (0)	1/1 (100)	1/1 (100)
*KCNQ2*	1	1/1 (100)	1/1 (100)	1/1 (100)	1/1 (100)	1/1 (100)
*KCNT1*	2	2/2 (100)	2/2 (100)	2/2 (100)	1/2 (50)	1/2 (50)
*KCTD3*	1	0/1 (0)	1/1 (100)	1/1 (100)	1/1 (100)	1/1 (100)
*NEXMIF*	1	1/1 (100)	1/1 (100)	1/1 (100)	0/1 (0)	0/1 (0)
*PAFAH1B1*	6	3/6 (50)	5/6 (83)	5/6 (83)	2/6 (33)	1/6 (17)
*PDHA1*	5	4/5 (80)	3/5 (60)	3/5 (60)	3/5 (60)	3/5 (60)
*PPP2R5D*	1	1/1 (100)	1/1 (100)	1/1 (100)	1/1 (100)	1/1 (100)
*SCN1A*	8	7/8 (88)	7/8 (88)	6/8 (75)	5/8 (63)	3/8 (38)
*SCN8A*	1	0/1 (0)	0/1 (0)	1/1 (100)	0/1 (0)	0/1 (0)
*SLC2A1* (*GLUT1*)	9	9/9 (100)	9/9 (100)	9/9 (100)	9/9 (100)	7/7 (100)^a^
*SLC12A5* (*KCC2*)	2	2/2 (100)	2/2 (100)	1/2 (50)	1/2 (50)	1/2 (50)
*SLC16A2*	1	1/1 (100)	1/1 (100)	1/1 (100)	1/1 (100)	^b^
*SLC25A12*	2	2/2 (100)	2/2 (100)	2/2 (100)	2/2 (100)	2/2 (100)
*SMC1A*	2	1/2 (50)	1/2 (50)	1/2 (50)	1/2 (50)	1/2 (50)
*STAMBP*	1	1/1 (100)	1/1 (100)	1/1 (100)	1/1 (100)	1/1 (100)
*STXBP1*	4	4/4 (100)	4/4 (100)	4/4 (100)	4/4 (100)	3/4 (75)
*STX1B*	2	1/2 (50)	1/2 (50)	0/2 (0)	0/2 (0)	0/2 (0)
*TSC1*	2	2/2 (100)	2/2 (100)	2/2 (100)	2/2 (100)	2/2 (100)
*TSC2*	6	3/6 (50)	2/6 (33)	2/6 (33)	1/6 (17)	1/6 (17)
*WDR62*	1	1/1 (100)	1/1 (100)	1/1 (100)	1/1 (100)	1/1 (100)
**Non-responders (*n* = 17 genes)**						
*ALG13*	1	0/1 (0)	0/1 (0)	0/1 (0)	0/1 (0)	0/1 (0)
*CACNA1E*	1	0/1 (0)	0/1 (0)	0/1 (0)	0/1 (0)	0/1 (0)
*CASK*	1	0/1 (0)	0/1 (0)	0/1 (0)	0/1 (0)	0/1 (0)
*CLN5*	1	1/1 (100)	0/1 (0)	0/1 (0)	0/1 (0)	0/1 (0)
*COL4A1*	2	0/2 (0)	0/2 (0)	0/2 (0)	0/2 (0)	0/2 (0)
*DYNC1H1*	2	0/2 (0)	0/2 (0)	0/2 (0)	0/2 (0)	0/2 (0)
*GLI3*	1	0/1 (0)	0/1 (0)	0/1 (0)	0/1 (0)	0/1 (0)
*MACF1*	1	1/1 (100)	0/1 (0)	0/1 (0)	0/1 (0)	0/1 (0)
*MAGEL2*	1	0/1 (0)	0/1 (0)	0/1 (0)	0/1 (0)	0/1 (0)
*NANS*	1	0/1 (0)	0/1 (0)	0/1 (0)	0/1 (0)	0/1 (0)
*NEMO/IKBKG*	1	0/1 (0)	0/1 (0)	0/1 (0)	0/1 (0)	0/1 (0)
*PCDH19*	1	0/1 (0)	0/1 (0)	0/1 (0)	0/1 (0)	0/1 (0)
*PIGT*	1	0/1 (0)	0/1 (0)	0/1 (0)	0/1 (0)	0/1 (0)
*PURA*	1	0/1 (0)	0/1 (0)	0/1 (0)	0/1 (0)	0/1 (0)
*SLC17A5*	1	0/1 (0)	0/1 (0)	0/1 (0)	0/1 (0)	0/1 (0)
*RORB*	2	2/2 (100)	0/2 (0)	0/2 (0)	0/2 (0)	0/2 (0)
*UFSP2*	1	0/1 (0)	0/1 (0)	0/1 (0)	0/1 (0)	0/1 (0)

Showing responders ≥ 50% and non-responders <50% seizure reduction to the KD. Number and percentages of responders in behaviour at 3 months, and seizure reduction (≥50%) at 3 and 6 months as well as at 1 and 2 years are given.

^a^Two patients not yet reached 2-year follow-up.

^b^One patient not yet reached 2-year follow-up.

**Table 6:** Structural rearrangements in responding and non-responding patients

Structural rearrangements	Patients *n*	Behaviour response 3 months; *n* (%)	Seizure response 3 months; *n* (%)	Seizure response 6 months; *n* (%)	Seizure response 1 year; *n* (%)	Seizure response 2 years; *n* (%)
**Responders**						
Del 2q24.3	1	1/1(100)	1/1 (100)	0/1 (0)	0/1 (0)	0/1 (0)
Del 5p	1	1/1 (100)	0/1 (0)	1/1 (100)	1/1 (100)	1/1 (100)
Del 9p	1	1/1 (100)	1/1 (100)	1/1 (100)	1/1 (100)	1/1 (100)
Del part X, dupl part 20	1	1/1 (100)	1/1 (100)	1/1 (100)	1/1 (100)	1/1 (100)
Dupl 5	1	1/1 (100)	1/1 (100)	1/1 (100)	1/1 (100)	1/1 (100)
Dupl 16p11.2	1	1/1 (100)	1/1 (100)	1/1 (100)	1/1 (100)	1/1 (100)
Parts tri 5p, mono 9p	1	1/1 (100)	1/1 (100)	1/1 (100)	0/1 (0)	0/1 (0)
Tri 15q, dup 12q	1	1/1 (100)	0/1 (0)	0/1 (0)	1/1 (100)	0/1 (0)
Trisomy 21	2	2/2 (100)	2/2 (100)	2/2 (100)	1/2 (50)	2/2 (100)
**Non-responders**						
Del 2q24.3	1	0/1 (0)	0/1 (0)	0/1 (0)	0/1 (0)	0/1 (0)
Del 16p region	1	0/1 (100)	0/1 (0)	0/1 (0)	0/1 (0)	0/1 (0)
Inv dup 15	1	1/1 (100)	0/1 (0)	0/1 (0)	0/1 (0)	0/1 (0)
Trisomy 13	1	0/1 (100)	0/1 (0)	0/1 (0)	0/1 (0)	0/1 (0)

Showing responders ≥ 50% and non-responders <50% seizure reduction to the KD.

Number and percentages of responders in behaviour at 3 months, and seizure reduction (≥50%) at 3 and 6 months as well as at 1 and 2 years are given.

Most disease-causing variants in both responders and non-responders were missense mutations, accounting for 25 out of 60 cases (41.7%) in responders and 20 out of 34 cases (58.8%) in non-responders (*P* = 0.11, OR = 0.5). Variants that introduced a stop codon into the reading frame, either due to an indel or nonsense mutation, were identified in 24 responders (40%) and nine non-responders (26.5%) (*P* = 0.19, OR = 0.99). Most deletions involved the loss of one to four exons within a single gene, observed in seven responders (11.7%) and four non-responders (11.8%) (*P* = 0.99, OR = 0.99).

### Causative variants and response to the ketogenic diet

The most common causative variants in responders were identified in *SLC2A1/GLUT1* (*n* = 9), *SCN1A* (*n* = 8), *PAFAH1B1* and *TSC2* (*n* = 6 each), *PDHA1* (*n* = 5), *STXBP1* (*n* = 4), *CDKL5* (*n* = 3) and in *DEPC5*, *GLDC*, *KCNT1*, *SLC12A5*, *SLC25A12*, *SMC1A*, *STX1B*, *TSC1* (*n* = 2 each) and in non-responders *COL4A1*, *DYNC1H1* and *RORB* (*n* = 2 each) (Table 5). The remaining 30 genes were each identified in only a single case. Children with variants in *SLC2A1* had a stable response over time where eight out of nine achieved seizure freedom, whereas children with variants in *PAFAH1B1* and *SCN1A* lost efficacy over time. Only one patient out of six with causative variants in *TSC2* was a responder at 2-year follow-up, contrary to the two with *TSC1* variant who responded well. Variants in *PDHA1* were found in five patients, three of whom became seizure free. A good response over the 2-year period was also seen in three out of four children with *STXBP1* variants. Two patients each with causative variants in *DEPDC5* and *GLDC* showed positive efficacy. Those carrying variants in *SLC25A12*, *KCNQ2* and *CSNK2A1* experienced seizure freedom (Table 5; Supplementary Table S3).

Patients with causative variants in *SLC2A1*, *SCN1A*, *STXBP1* and *PAFAH1B1* showed a significantly better response to KD than expected by chance given the size of the respective group compared to the mean response for all (*P* < 0.001, *P* = 0.007, *P =* 0.023 and *P* = 0.036).

The aetiologies *COL4A1*, *DYNC1H1* and *RORB*, each with two patients, showed no seizure reduction in response to the diet. However, the patients with *RORB* aetiology showed cognitive improvement.

Additionally, structural rearrangements that include large chromosomal aberrations, were found in 14 patients, with 10 showing a positive response to KD (*P* = 0.014) (Table 6; Supplementary Table S3). Notably, four patients including two boys with trisomy 21 were seizure free after 2 years on diet.

### Functional pathways and response to ketogenic diet (gene groups)

Many known genes associated with epilepsy can be classified into functional groups, as demonstrated by Guerrini *et al*.^21^ The 48 causative genes in our cohort were grouped into functional groups and analysed regarding seizure response (Table 7). Statistical comparison of the functional groups showed that causative variants in genes coding for transporters had a significantly better response (*P* = 0.009, RR = 1.53, 95% CI 1.14–2.05) while genetic variants coding for proteins in the cell structural integrity and/or homeostasis group had significantly worse response (*P* = 0.00006, RR = 0.0, 95% CI NA-0.29). The transporters included variants in *SLC2A1*, *SLC12A5*, *SLC25A12*, *SLC16A2* and *SLC17A5*. Good responses were also seen in children carrying variants in ion channels, particularly sodium (*SCN1A*) and potassium channels (*KCNQ2*, *KCNT1*, *KCNA2* and *KCTD3*) with response in 13/15 = 86.7% (*P* = 0.050) as well as in those carrying variants in neurotransmitter receptors GABA-A (*GABRB3*) and NMDA (*GRIN1* and *GRIN2D*) with response in *3/3 = 100%;* (*P* = 0.091).

**Table 7:** Grouping of genes containing causative variants into functional groups and response to the KD

			Response rate		RR (95% CI) Univariate	
Encoded protein function	Sub-groups	Causative genes	n	%		*P*-value
**Neurotransmitter receptors**	GABA-A receptor NMDA receptor	*GABRB3* *GRIN1*, *GRIN2D*	3/3	100	1.64 (0.85–2.15)	0.091
**Transporters**		*SLC2A1*, *SLC12A5*, *SLC16A2*, *SLC25A12*, *SLC17A5*^a^	14/15	93	1.53 (1.14–2.05)	**0**.0094*
**Ion channels**	Sodium channel	*SCN1A*, *SCN8A*	13/15	87	1.42 (1.00–1.94)	0.050
	Potassium channel	*KCNA*, *KCNT1*, *KCNQ2*, *KCTD3*				
	Calcium channel	*CACNA1E* ^a^				
**Synapse related**		*STX1B*, *STXBP1*, *CASK*^a^	5/7	71	1.17 (0.56–1.78)	0.59
**Energy homeostasis and mitochondrial function**		*FARS2*, *PDHA1*	4/6	67	1.10 (0.45–1.74)	0.78
**Cell growth, division and proliferation-related**	Gene regulation, DNA integrity chromatin, and RNA binding Neuronal proliferation, differentiation and/or migration	*CDKL5*, *SMC1A* *BRAF*, *NEXMIF*, *PAFAH1B1*, *PPP2R5D*, *WDR62*, *PURA*^a^, *RORB*^a^	19/32	59	0.98 (0.66–1.40)	0.89
	P13/AKT/mTOR pathway and signalling	*CSNK2A1*, *DEPDC5*, *STAMBP*, *TSC1*, *TSC2*, *GLI3*^a^, *NEMO/IKBKG*^a^				
**Metabolism-related**		*ADSL*, *GLDC*, *ALG13*^a^, *NANS*^a^	2/5	40	0.66 (0.13–1.41)	0.37
**Cell structural integrity and/or homeostasis**	Intraneuronal transport and trafficking Protein biosynthesis/degradation- related	*CLN5* ^a^, *DYNC1H1*^a^ *MAGEL2*^a^, *PIGT*^a^, *UFSP2*^a^	0/10	0	0.00 (NA-0.29)	**0**.000062*
	Cytoskeleton, extracellular matrix and cell–cell interaction	*COL4A1* ^a^, *MACF1*^a^, *PCDH19*^a^				

Totally, 93 patients with causative variants in 48 genes listed in eight protein functional groups. Number of responders (≥50% seizure reduction) per total number of individuals at 3 months on the KD. Group 2 with no causative genetic findings, was used as reference. Number of patients per causative gene is shown in Table 5. Causative genes (*n* = 31) in responders and (*n* = 17) in non-responders.

^a^Marks genes in non-responders.

^*^Significant differences (*P* < 0.05) given in bold.

In addition, favourable responses were seen among children with epilepsy due to variants in synapse-related genes (*5/7 = 71%*) including *STXBP1*, *STX1B* and cell growth, division and proliferation-related genes (*19/32 = 59%*) including *SMC1A* and mTOR pathway genes *DEPDC5*, *BRAF*, *TSC1* and *TSC2.* In contrast, children carrying variants in genes related to cell structural integrity (*DYNC1H1*), extracellular matrix (*COL4A1*), protein biosynthesis and degradation-related (*UFSP2*, *MAGEL2*, *PIGT*) were non-responders (*P* < 0.05).

### Pathogenic findings in whole genome sequencing research setting

After obtaining signed informed consent from families, we proceeded to examine the entire genomes, in 21 cases. In one case, we found a *de novo* missense mutation in *CSNK2A1*, a gene not included in the epilepsy panel at the time. The patient is a boy with severe developmental delay and myoclonic epilepsy who became seizure free on KD. The *CSNK2A1* gene is linked to Okur-Chung neurodevelopmental syndrome in OMIM. The phenotype partially matched our patient. Literature search showed several cases of *CSNK2A1* patients with global developmental delay and various types of seizures.^22^ In another case, a rare *de novo* deletion in *HDAC9* was revealed, a gene where no patients were reported but the variant itself and the gene function was of large interest. It was uploaded to Matchmaker Exchange where we instantly found cases in other clinical settings who matched phenotypically with our patient. A collaboration to perform functional studies in *HDAC9* variant carriers was started (studies ongoing).

### Variants of uncertain significance

The WGS analysis unveiled 18 VUS (variant of uncertain significance) candidates across 17 patients. The candidate variants grouped based on their protein function into ion channels (*SCN1A* and *CACNA1A*), synapse related (*SHANK1*, *WDR7* and *TMEM184B*), cell growth, division and proliferation-related (*TET1*, *SMG6*, *BICRA*, *HDAC9*, *A2BP1*, *PTCH1*, *DEPDC5* and *PIK3R2*), cell structural integrity and/or homeostasis (*ARMCX4*, *HSP90AA1*, *TRIP12* and *LIN7B*) and in energy homeostasis and mitochondrial function (*POLG*).

## Discussion

We present a large cohort study of all 226 children treated with the classic KD for at least 3 months due to epilepsy during the last 25 years at our centre. The aim was to evaluate seizure response in relation to causative genetic findings in order to identify prognostic genetic markers for efficacy of the diet. All 226 children had at least 1-year follow-up and 216 had a 2-year follow-up (96%). Our cohort is demographically similar to other large KD cohorts concerning age at KD start and at epilepsy onset, previous trials of ASMs and percentage of ID.^2,23,24^ Compared with the study of Lambrechts *et al*.,^4^ the epilepsy onset was earlier and ID more frequent.

The KD seizure response in our cohort was in line with three large open prospective and retrospective studies with 12 months follow-up.^2,23,24^ Two randomized controlled studies, which included all started patients with follow-up at 3 months had a lower response rate of 38%^3^ and 50%,^4^ probably in part due to a high number of non-starters or withdrawers counted as non-responders. A meta-analysis of five RCTs showed a KD response rate of 35–56% at 3–4 months.^25^ In our study, we included only patients that were still on KD at 3 months as this time is considered appropriate for KD efficacy evaluation in the consensus report.^26^

In our study, seizure response was not related to age at diet start or gender which is in line with previous studies.^2,23,24^ However, we found efficacy to be related to age of epilepsy onset with a better seizure reduction the younger the patient at onset. The study by Kang *et al*.^24^ did not find a correlation between age at onset and response, which may be due to an earlier onset age in our cohort. In our study, the epilepsy type affected response rate. Responders had significantly less often combined epilepsy compared to non-responders and proportionally more focal epilepsy however the latter was non-significant. A previous study could not find differences in seizure control between focal and generalized epilepsies^24^ but studies relating combined epilepsy type and KD response are lacking. Regarding specific seizure types, after adjusting for confounders, we found no differences related to response which agrees with earlier studies.^2,23,24^

The ketone body β-OHB is involved in numerous pathways in the CNS including provision of substrate for the TCA cycle, and evidence is increasing for its role in modulation of KATP channels, acting as endogenous ligands for free fatty acid receptors, inhibition of histone deacetylation, and anti-inflammatory effects.^27^ Preclinical experimental studies have pointed to a role of ketones in the anti-seizure effect of KD but clinical studies on correlation of β-OHB blood levels and seizure control have not shown consistent results.^28^ In our study, fasting blood β-OHB levels at 3 months in 96% of the cohort showed similar levels in responders and non-responders. Thus, our results do not provide support for peripheral blood β-OHB levels as a biomarker of KD efficacy.

Giving formula-based KD products in a gastrostomy has been proposed to be an advantage in KD treatment by reducing practical issues. It was reported to give a good compliance and efficacy in a small study.^29^ However, our 69 children on KD fed by gastrostomy did not differ in seizure response.

In the 73 patients without genetic aetiology revealed and not genetically tested with WES/WGS, a probable non-genetic aetiology to the epilepsy was found in 45%. A proportionally good response was seen in those with epilepsy due to HIE and prematurity but less response after encephalitis, which is in line with an Australian study reporting good efficacy in acquired structural aetiology as HIE and prematurity with brain injury.^30^

Apart from seizures, improvement in behaviour was noted at 3 months in a majority of children, mostly concerning communication and contact. A review on cognition and KD with objective neuropsychological testing showed that alertness was improved but not global cognition concluding that this was not only due to seizure reduction but also a direct effect of KD.^31^ Interestingly, in the clinic we observed increased alertness and improved behaviour even in children without seizure reduction and a some of them continued KD.

Of the 153 genetically tested patients, 107 received a causative genetic diagnosis (69.9%). In line with previous studies a causative genetic finding was associated with an early onset of epilepsy.^32-34^ Although seizure response to KD in relation to whether a causative finding was found or not did not reach significance at ≥50% seizure reduction level, it did reach significance in those with >90% seizure reduction at 1 and 2 years-follow-up and in seizure free at 6 months and 1 year. Also, KD efficacy was more sustained with time in those with a confirmed genetic aetiology. Our findings conform with two previous smaller studies showing a better seizure response in cases with confirmed genetic aetiology.^35,36^

The increase of identified monogenic epilepsies has led to a surge for precision therapies based on understanding of the underlying disease mechanisms. Beneficial effects of KD has long been shown in some genetic neurometabolic disorders with impaired energy production in which epilepsy is common.^5^ In recent years, studies have shown that some specific genetic aetiologies respond well to KD but others have less efficacy.^12^ In our study, 31 out of 48 genes with causative pathogenic variants were associated with KD response. Children with variants in *SLC2A1*, *SCN1A*, *STXBP1* and *PAFAH1B1* had highly significant seizure response (*P* < 0.05). Our results in Glut1 DS caused by variants in *SLC2A1* with seizure freedom at 1 year in 8/9 cases adds to previous evidence of KD efficacy in this disorder.^37^ In our cohort of *SCN1A* variants, 7/9 (78%) were responders at 3 months and 3/9 (33%) at 2 years. This is in line with data from the Mayo clinic, which report 64% and 45% KD responders at this time points.^13^ The *STXBP1* gene encodes a syntaxin-binding protein associated with synaptic vesicle release. Our four children with causative variants in *STXBP1* were responders including one seizure free. Previous studies on *STXBP1* variants have shown diverging results.^11,12,38,39^ Five out of six children with lissencephaly and causative variants in *PAFAH1B1* in our cohort showed KD response at 3 and 6 months, and at 2-year follow-up, one was still a responder. Our results are in line with an Australian study, which report a good KD response in lissencephaly in 3/4 patients at 3 months and in 2/3 at 12 months.^40^

Not reported previously, to our knowledge, we found a good, although not significant, KD response related to causative variants in *CSNK2A1*, *FARS2*, *GABRB3*, *GRIN1*, *KCTD3*, *SLC16A2* and *STX1B* but a lack of response for causative variants in *CLN5*, *GLI3*, *MACF1*, *MAGEL2*, *NANS*, *NEMO/IKBKG*, *SLC17A5, RORB* and *UFSP2.*

To optimize the use of KD in precision medicine, knowledge on the pathogenesis of epilepsy and mechanisms of action of KD are needed. Here, not only the seizure response for specific gene variants may prove useful but also efficacy for functional groups of genes targeting specific functions of neurons or networks. Recently, studies approaching this question are published with categorization of causative variants in relation to KD response in children with refractory epilepsy.^36,41,42^ The ion channel group was studied by Muthaffar *et al.*,^36^ showing good KD efficacy but too small numbers for statistical conclusions, and by Song *et al*.,^42^ showing correlations with seizure freedom. On the other hand, a lower response in the channelopathy group was found by Kim *et al*.^41^ Interestingly, we found a good efficacy in this group with 87% responders at 3 months, almost reaching significance (*P* = 0.050). However, the gene group categorizations are not equivalent between these studies, and they can thus not be fully compared. In the future, agreement on categorizations and larger study groups would be beneficial.

The functional gene group with the best (93.3%) and highly significant response in our cohort was the transporter group including *SLC2A1*, *SLC12A5*, *SLC16A2*, *SLC17A5* and *SLC25A12* genes. *SLC2A1* is discussed above and variants in another transporter gene, *SLC25A12* cause AGC1 deficiency which is associated with a severe neurological phenotype with hypomyelination and epilepsy.^43^ We published the first case of AGC1 deficiency in a patient that started KD at 6 years.^6^ Our second patient with an early diagnosis of AGC1 deficiency started KD at 5 months. In both cases, seizures disappeared rapidly after KD start but reappeared later in a milder form. Cognitive and motor function did improve but a severe neurological dysfunction persists, however, with a better outcome in the patient with an early KD start. Bölsterli *et al.* describes 14 patients with AGC1 deficiency with the most prominent effect of KD on reducing seizures.^7^ Their results are in line with ours including findings of increased MRI myelination and increased NAA ratio on MRS. Pathogenic variants in the transporter gene *SLC12A5* also result in early onset epilepsy with refractory seizures and delayed development.^44^ Our two cases were both responders at 3 months but one lost efficacy later while in the other response persisted for 2 years.

Patients in our cohort with variants in sodium (including *SCN1A*) and potassium channels showed good KD response. Modulation of potassium channels has been proposed as a possible mechanism of action of KD.^5^ Our five patients with variants in potassium channel encoding genes were KD responders. One patient had a causative variant in *KCNQ2* with seizure onset at 6 months, daily tonic and focal seizures and a mild ID. The child became seizure free soon after KD start and remains so after KD termination and >5 years follow-up. Our results agrees with a Korean study in which five out of six patients with *KCNQ2* variants were KD responders.^12^ Another patient with a causative variant in *KCNA2* had focal seizures, multi-focal epileptiform discharges and mild ID, which correlates to a milder phenotype described.^45^ KD gave seizure reduction and improved behaviour. To our knowledge, this gene has not been reported previously concerning KD response. The *KCNT1* gene is associated with an early onset refractory epilepsy.^46^ Our two patients with causative variants in this gene and a typical phenotype both responded to KD at 6 months and one of them still at the 2-year follow-up. These results agree with a study of *KCNT1* variants in which almost half of 23 cases were KD responders.^47^ Variants in *KCTD3* are associated with developmental epileptic encephalopathy.^48^ Our patient had severe global delay, brain malformations and early onset refractory epilepsy. The child was a KD responder and to our knowledge KD response has not been reported previously.

Our results of KD related to neurotransmitter receptor gene variants are promising with the three patients being KD responders. *GRIN1* and *GRIN2D* code for subunits in the NMDA (N-methyl-D-aspartate) receptors and pathological variants are associated with a severe neurological phenotype including epilepsy. Our study included one patient each with causative variants in these genes and, interestingly, both had a > 90% seizure reduction all through the 2-year follow-up. A case report of one child with *GRIN2D* variant failed KD.^49^ Data on *GRIN1* and KD response are lacking. Our patient with a causative variant in *GABRB3*, encoding a subunit of the GABA receptor, became seizure free while previous reports are lacking.

In our study, 17 out of 48 genes with causative pathogenic variants were not associated with KD response. None of the patients with gene variants belonging to the structural integrity and/or homeostasis functional group responded to KD. Among other genes, the group included *DYNC1H1* and *COL4A1* gene variants with two participants each who exhibited typical phenotypes. Previously, KD response has been reported in one patient with *DYNC1H1*^50^ variant and in one out of two with *COL4A1* variants.^12^ Genotype-phenotype descriptions for *DYNC1H1* and *COL4A1* are found in Supplementary Materials as well as data on mTOR genes, *GLDC* and *CDKL5.*

Several of the patients with structural chromosomal aberrations did have a favourable KD response. Particularly interesting are the two children with trisomy 21 and ACTH resistant epileptic spasms who both were seizure free throughout the 2-year study period, in line with a case report with trisomy 21 and epileptic spasms.^51^

Limitations of our study include relying on seizure diaries registered by caregivers and not having objective long-term EEG registrations on seizure frequencies. However, the same carers registered seizures before and during KD and patient-reported seizure diaries are the common outcome measure in research studies. We did not include generalized non-motor seizures as they are difficult to count reliably. Also, data were compiled retrospectively from medical records, but we followed a structured evaluation protocol, and only a few doctors were responsible for all patients. Another limitation is not having WES/WGS data on the entire cohort as these methods were not available at study start. Strengths to mitigate these limitations are the large patient cohort and an almost complete long-term follow-up of included children. Being an epilepsy referral centre may imply a possible selection bias of more complex epilepsy cases and a large majority of our patients were of European origin why our results may not be directly generalizable to broader patient groups.

Despite its long use and satisfying efficacy, the mechanism of action by which the KD suppresses seizures remains unknown in the majority of cases. Several hypotheses have been proposed implying e.g. direct anticonvulsant eﬀects of ketone bodies or polyunsaturated fatty acids.^52,53^ Alterations in electrolyte and water balance, brain pH, critical amino acids, neurotransmitters and inflammatory cytokines have been implicated, together with epigenetic changes affecting gene expression. Decreased glucose levels, reduced oxidative stress, alterations in brain energy reserve and a shift in energy metabolism from glycolytic to oxidative phosphorylation have also been proposed. Direct membrane effects and channel modiﬁcations are among other suggested explanations. Taken together, it seems likely that the KD acts through several different mechanisms, and that it exerts its effects through different mechanisms in different patients.

## Conclusion

Our study supports that genetic aetiologies can predict efficacy of KD in epilepsy. By using WES/WGS, we increased the genetic aetiologies to include 48 different genes in 107 of 226 children with drug-resistant epilepsy. Overall, KD is an efficient treatment in genetic epilepsies. Some genes and gene groups seem to respond better to KD than others. *SLC2A1*, *SCN1A*, *STXBP1* and *PAFAH1B1* were associated with highly significant responses. We found positive KD efficacy in patients with variants in genes encoding three different groups of membrane-related proteins; transporters, ion channels and neurotransmitter receptors, while genes involving cell structural integrity and/or homeostasis were non-responders. Genetic variants are promising predictors of KD efficacy, but further studies are needed and may also help elucidate the mechanisms of action of KD.

## Supplementary Material

fcaf134_Supplementary_Data

## Data Availability

The data that support the findings of this study are available on request from the corresponding author. The data are not publicly available due to privacy or ethical restrictions.
