# Supplementary material for: Genetic aetiologies in relation to response to the ketogenic diet in 226 children with epilepsy
Source: Brain Commun. 2025 Apr 5;7(2):fcaf134. doi: 10.1093/braincomms/fcaf134 (PMC12022961; doi:10.1093/braincomms/fcaf134)
Supplement: fcaf134_Supplementary_Data [file fcaf134_supplementary_data.zip › Supplementary_Materials.pdf]

Supplementary Materials

Study design

The diet treatment was carried through by our KD team consisting of a senior neuropediatrician, a trained dietitian and an epilepsy nurse. The seizure response was determined from seizure calendars. Before diet start the parents and caregivers were given seizure calendars used routinely in our clinic and were instructed to make daily notes on the number and types of seizures of their child. The mean seizure frequency during the month before starting the KD was taken as baseline and was compared with the mean frequency the month before each follow-up visit at 3 and 6 months as well as at 1 and 2 years after diet start. Children with less than 50% seizure reduction were considered non-responders, and those with a seizure reduction of 50% or more were considered responders.

The majority of children had been evaluated concerning their intellectual level by a neuropsychologist before diet start with use of standard methods appropriate for age and performance of the child. Children with motor dysfunctions were assessed by a physiotherapist and often also an occupational therapist. Diagnosis on ID and motor dysfunction were confirmed in agreement with the responsible neuropediatrician. In young children, not yet tested at diet start, demographic data on intellectual function was retrieved at a later timepoint when neuropsychological testing had been performed.

Evaluation of behavior was done before diet start as well as at follow-up after 3 months on diet. Usually, the same senior neuropediatrician made a clinical examination of the patient and had a structured interview with the parents including opinions from caregivers at preschool or school. Questions included the child´s level of alertness, eye contact, social interest and interaction, attention, verbal, and motor function. The result of the evaluation was a summary of the parent’s perception of the child’s behavior and the clinical examination of the neuropediatrician. The behavior scale was considered improved, unchanged or worsened. It was considered improved if parents and doctor considered changes to be of clear importance for the function of the child.

To initiate and maintain the KD, we followed a standardized protocol for the classic KD, with a slightly modified version of the protocol from the Johns Hopkins Hospital.[1] Our KD protocol has developed in some aspects over the years, mainly concerning fasting and ratio at diet start.

During the first years of the study, fasting during the first 24 h was used followed by gradual introduction of the KD meals. Thereafter, we stopped fasting and gave full ketogenic diet meals from diet start. Also, during the first years of the study, most children started KD on a 4:1 ratio of fat to protein and carbohydrates except for the children under 2 years of age, who were started on a 3:1 ratio. During later years, children are started on a ratio between 2:1 and 3:1 and the ratio is gradually increased in half steps i.e. after one week of 2:1 it was increased to 2.5:1. Usually an optimal ratio for the individual child is reached within 3-6 weeks and is kept unchanged until the follow-up visit at 3 months. When possible, children continued with unchanged ASMs in type and dose from start to follow-up of KD at 3 months to minimize confounding factors.

A dietitian, specially trained to carry out KD treatment, calculated the total calorie level per day and the composition of all meals and supplements for the individual child. The calculation of the calorie amount was based on a 2-day food diary filled out by the parents which was modified by the recommended calorie amount according to the child´s age and level of physical activity. In the composition of the individual meals, the child´s food preferences were considered as much as possible. A minimum of 1g/kg body weight per day of protein was included. Fish oil, 4-8 g per day, was given together with the meals. All children were supplemented with multivitamins and minerals, including potassium citrate, calcium, magnesium, zinc, and selenium, and were provided with 100 mg/kg/day of carnitine.

To initiate the diet, the child was hospitalized during a 5-day stay to introduce the meals, monitor potential side effects, carry out an educational program for the family on various aspects of the treatment and to give practical instructions in how to prepare the meals. Before diet start and at follow-up visits, venous blood sampling was done in the morning after an overnight fast for an extensive laboratory testing including the ketone beta-hydroxybutyrate (β-OHB). At discharge a scale and often a blood ketone monitor were taken home. After discharge, the diet was fine-tuned by frequent telephone consultations with the family with monitoring of efficacy, blood ketone levels and weight. The KD ratio was successively increased and the number of calories adjusted in order to achieve an optimal seizure control. At follow-up visit after 3 months´ evaluation was made of seizure response and behavior, and it was decided whether to continue or taper the diet. If continuing diet follow-up outpatient visits were held after 6 months and after 1 and 2 years.

Whole Genome Sequencing

Sequencing was performed on the Illumina nova-Seq 6000 with a median coverage of 30x. The raw WGS data was processed using the Mutation Identification Pipeline (MIP): a bioinformatic pipeline composed of custom-developed open-source tools, as well as pre-existing tools, for variant calling, annotation and prioritization.[2] MIP is continually undergoing development and currently detects single nucleotide variants, insertion/deletions, structural variants, uniparental disomy, repeat expansions and copy number variants. MIP generates a rank score for each variant which summates multiple parameters including Mendelian inheritance pattern, rarity, conservation, and predicted effect on protein. The rank score enables prioritization of WGS data to a manageable set of potentially disease-causing variants. The prioritized WGS data is uploaded to Scout: a user-friendly web browser-based interface which enables the analysis and sharing of WGS data by multidisciplinary teams focusing on different disease groups.

In the clinical analysis the genome data in Scout is filtered in silico by using clinically relevant gene panel(s) such as the epilepsy-panel with 550 genes (version 21) or the inborn errors of metabolism panel including 1070 genes (version 21). The gene panels are compiled by teams with expertise in the specific area and are updated 2-4 times per year. Panels can also be customized by adding human phenotype ontology (HPO) terms specific for each patient. If no causative variants are found in the clinical setting, we have the option to expand the analysis by removing filters and accessing the whole genome.

Genes

mTOR signal pathway genes The KD has been shown to attenuate the mTOR signal pathway.[3] Many genes that encode proteins in this pathway have implications in epilepsy. The *DEPDC5* gene encodes a subunit in the protein GATOR1 complex, and variants can cause both lesional and non-lesional focal epilepsy.[4] Our two patients with *DEPDC5* variants were both responders to KD. Similar results were seen in a Polish study in which their three patients got seizure free.[5] However, in a Korean study, three patients with DEPDC5 variants were KD non-responders.[6] Efficacy of KD in variants in the TSC genes has been shown in several studies, lately in a study from China of 53 children with TSC.[7] Here 47.1% were responders after 3 months but no difference was seen between *TSC1* or *TSC2* genes. In our cohort, 2/2 children with variants in *TSC1* gene but in 1/6 with *TSC2* variants were KD responders after 2 years.

GLDC Pathogenic variants in *GLDC* gene results in NKH which is an autosomal recessive disorder of glycine metabolism which accumulates in the brain. Glycine is a neurotransmitter and exerts excitatory effects in the cerebral cortex. Excess levels can cause epilepsy and hypotonia. KD was reported to reduce early onset ASM-resistant myoclonic seizures in three NKH patients.[8] Other case reports confirm this finding.[9,10] The seizure reduction in our two patients with neonatal onset myoclonic epilepsy supports earlier findings.

CDKL5 Concerning our three patients with *CDKL5* mutation, two were non-responders but one had a >90% seizure reduction for more than 2 years. In a single center study, 5/10 patients with *CDKL5* mutation were KD responders at 2 years while four responded at earlier timepoints.[11] In a multicenter study on patients with *CDKL5* mutation, 2/12 (17%) were KD responders for >6 months.[12]

DYNC1H1 The *DYNC1H1* (human dynein cytoplasmic 1 heavy chain 1) gene encodes a large subunit of the cytoplasmic dynein complex. Refractory epilepsy and ID are typical features. One patient with a pathogenic DYNC1H1 variant was reported to have a 50% seizure reduction but seizures later returned.[13] In our two cases, neither child had any KD seizure response.

The gene ***COL4A1*** (collagen type IV alpha 1 chain) encodes a chain of collagen which is a component in the vascular basement membranes. *COL4A1* mutations typically show a multisystemic phenotype, including epilepsy, ID and microcephaly. Our two patients, with a typical phenotype of *COL4A1*, were non-responders to KD. Response to KD was reported in one out of two patients with COL4A1 variants.[14]

References

1. Swink TD, Vining EP, Freeman JM. The ketogenic diet: 1997. *Adv Pediatr*. 1997;44:297-329.

2. Stranneheim H, Lagerstedt-Robinson K, Magnusson M, *et al*. Integration of whole genome sequencing into a healthcare setting: high diagnostic rates across multiple clinical entities in 3219 rare disease patients. *Genome Med*. Mar 17 2021;13(1):40. doi:10.1186/s13073-021-00855-5

3. Schubert-Bast S, Strzelczyk A. Review of the treatment options for epilepsy in tuberous sclerosis complex: towards precision medicine. *Ther Adv Neurol Disord*. 2021;14:17562864211031100. doi:10.1177/17562864211031100

4. Myers KA, Scheffer IE. DEPDC5 as a potential therapeutic target for epilepsy. *Expert Opin Ther Targets*. Jun 2017;21(6):591-600. doi:10.1080/14728222.2017.1316715

5. Winczewska-Wiktor A, Hirschfeld AS, Badura-Stronka M, Komasińska-Piotrowska P, Steinborn B. Analysis of Factors That May Affect the Effectiveness of Ketogenic

Diet Treatment in Pediatric and Adolescent Patients. *J Clin Med*. Jan 25 2022;11(3)doi:10.3390/jcm11030606

6. Lee S, Kim SH, Kim B, *et al*. Genetic diagnosis and clinical characteristics by etiological classification in early-onset epileptic encephalopathy with burst suppression pattern. *Epilepsy Res*. Jul 2020;163:106323. doi:10.1016/j.eplepsyres.2020.106323

7. Fang Y, Li D, Wang M, *et al*. Ketogenic Diet Therapy for Drug-Resistant Epilepsy and Cognitive Impairment in Children With Tuberous Sclerosis Complex. *Front Neurol*. 2022;13:863826. doi:10.3389/fneur.2022.863826

8. Cusmai R, Martinelli D, Moavero R, *et al*. Ketogenic diet in early myoclonic encephalopathy due to non ketotic hyperglycinemia. *Eur J Paediatr Neurol*. Sep 2012;16(5):509-13. doi:10.1016/j.ejpn.2011.12.015

9. Nickerson SL, Balasubramaniam S, Dryland PA, *et al*. Two Novel GLDC Mutations in a Neonate with Nonketotic Hyperglycinemia. *J Pediatr Genet*. Sep 2016;5(3):174-80. doi:10.1055/s-0036-1584358

10. Ning JJ, Li F, Li SQ. Clinical and genetic analysis of nonketotic hyperglycinemia: A case report. *World J Clin Cases*. Aug 6 2022;10(22):7982-7988. doi:10.12998/wjcc.v10.i22.7982

11. Jagadish S, Payne ET, Wong-Kisiel L, Nickels KC, Eckert S, Wirrell EC. The Ketogenic and Modified Atkins Diet Therapy for Children With Refractory Epilepsy of Genetic Etiology. *Pediatr Neurol*. May 2019;94:32-37. doi:10.1016/j.pediatrneurol.2018.12.012

12. Müller A, Helbig I, Jansen C, *et al*. Retrospective evaluation of low long-term efficacy of antiepileptic drugs and ketogenic diet in 39 patients with CDKL5-related epilepsy. *Eur J Paediatr Neurol*. Jan 2016;20(1):147-51. doi:10.1016/j.ejpn.2015.09.001

13. Su T, Yan Y, Hu Q, Liu Y, Xu S. De novo DYNC1H1 mutation causes infantile developmental and epileptic encephalopathy with brain malformations. *Mol Genet Genomic Med*. Mar 2022;10(3):e1874. doi:10.1002/mgg3.1874

14. Ko A, Jung DE, Kim SH, *et al*. The Efficacy of Ketogenic Diet for Specific Genetic Mutation in Developmental and Epileptic Encephalopathy. *Front Neurol*. 2018;9:530. doi:10.3389/fneur.2018.00530
